# Reliable Fabrication of Mineral‐Graded Scaffolds by Spin‐Coating and Laser Machining for Use in Tendon‐to‐Bone Insertion Repair

**DOI:** 10.1002/adhm.202402531

**Published:** 2024-08-05

**Authors:** Yidan Chen, Min Hao, Ismael Bousso, Stavros Thomopoulos, Younan Xia

**Affiliations:** ^1^ School of Materials Science and Engineering Georgia Institute of Technology Atlanta GA 30332 USA; ^2^ The Wallace H. Coulter Department of Biomedical Engineering Georgia Institute of Technology and Emory University Atlanta GA 30332 USA; ^3^ Department of Orthopedic Surgery Department of Biomedical Engineering Columbia University New York NY 10032 USA; ^4^ School of Chemistry and Biochemistry Georgia Institute of Technology Atlanta GA 30332 USA

**Keywords:** hydroxyapatite nanoparticles, laser machining, mineral gradient, spin‐coating, tendon enthesis

## Abstract

A reliable method for fabricating biomimetic scaffolds with a controllable mineral gradient to facilitate the surgical repair of tendon‐to‐bone injuries and the regeneration of the enthesis is reported. The gradient in mineral content is created by sequentially spin‐coating with hydroxyapatite/poly(ε‐caprolactone) suspensions containing hydroxyapatite nanoparticles in decreasing concentrations. To produce pores and facilitate cell infiltration, the spin‐coated film is released and patterned with an array of funnel‐shaped microchannels by laser machining. The unique design provided both mechanical (i.e., substrate stiffness) and biochemical (e.g., hydroxyapatite content) cues to spatially control the graded differentiation of mesenchymal stem cells. Immunocytochemical analysis of human mesenchymal stem cell‐seeded scaffolds after 14 days of culture demonstrated the formation of a spatial phenotypic cell gradient from osteoblasts to mineralized chondrocytes based on the level of mineralization in the scaffold. By successfully recreating compositional and cellular features of the native tendon enthesis, the biomimetic scaffolds offer a promising avenue for improved tendon‐to‐bone repair.

## Graphical abstract

A functionally‐graded scaffold with a well‐controlled mineral gradient is fabricated using a layer‐by‐layer spin‐coating method. Patterned with an array of funnel‐shaped microchannels by laser machining, the scaffold promotes stem cell infiltration and graded differentiation according to the level of mineralization. Successfully recreating the architectural and cellular features of native enthesis, the scaffold shows great promise in augmenting connective tissue repair.

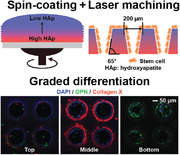

## 1 Introduction

The enthesis, a specialized fibrocartilaginous tissue that connects a tendon (or a ligament) to a bone, is responsible for transmitting mechanical loads between the tendon and the bone during joint motion. For musculoskeletal injuries occurring at or near this soft‐to‐hard tissue interface, it has been a challenge to regenerate the transitional tissue and thereby restore the function,^[^ ^1^ ^]^ resulting in high failure rates after surgical repair procedures.^[^ ^2^ ^]^ In particular, critical features, including a spatially graded composition and hierarchical structure of the extracellular matrix and a unique population of cells with a phenotypic gradient, are not recreated during the healing process.

A promising strategy for addressing this clinical challenge involves the use of biomimetic scaffolds with mineral gradients to promote the regrowth of the functionally graded enthesis.^[^ ^1c^ ^]^ These scaffolds not only enable a smooth transition in mechanical properties between the tendon and bone but also induce a spatial gradient in cell phenotype to match the features of native enthesis by offering both biochemical and mechanical cues through, for example, hydroxyapatite (HAp) content and corresponding substrate stiffness, respectively.^[^ ^3^ ^]^

There are several reports on the fabrication of mineral‐graded biomimetic scaffolds. For instance, Splazzi et al. proposed a triphasic scaffold made of poly(lactic‐co‐glycolic) acid and bioactive glass to promote zone‐specific distribution of cells in vitro and the development of fibrocartilage‐like tissue in vivo.^[^ ^4^ ^]^ More recently, Bai et al. 3D‐printed living enthesis‐like tissues embedded with layer‐specific growth factors to drive human umbilical cord mesenchymal stem cells toward a fibrocartilaginous‐like structure.^[^ ^5^ ^]^ As a critical limitation for future translation, it is challenging to functionally integrate these scaffolds with the existing musculoskeletal system. Many of the scaffolds suffer from the mismatch between the length scale of the artificial gradient and that of the native enthesis,^[^ ^6^ ^]^ as well as a discrete or abrupt transition in mineral content that is prone to mechanical failure.^[^ ^4^, ^7^ ^]^ Other approaches, such as brush coating,^[^ ^8^ ^]^ have limited scalability due to the intrinsically low batch‐to‐batch consistency when manual brushing is involved. Recently, our group demonstrated a method for fabricating poly(ε‐caprolactone) (PCL) scaffolds with a mineral gradient based upon swelling‐induced diffusion.^[^ ^9^ ^]^ While this method is straightforward and compatible with scaled‐up production, it still suffers a few drawbacks. First, although the length scale of the mineral gradient can be tuned by varying the extent of polymer swelling, the steepness of the gradient is not easily adjustable with passive diffusion as the main driving force. Second, the graded zone only exists in the middle of the scaffold, with relatively thick nongraded regions on both ends, presumably due to the need to have enough driving force for the polymer chains to diffuse.

**Figure 1:**
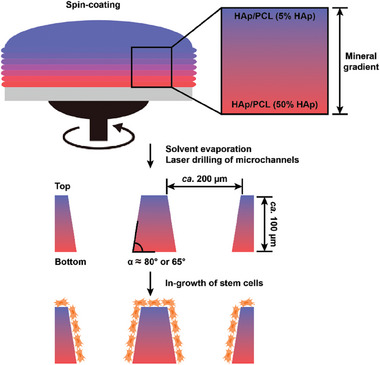
Schematic showing the fabrication of a scaffold containing a vertical gradient in mineral content through layer‐by‐layer spin‐coating with PCL solutions containing HAp nanoparticles at decreasing concentrations, followed by laser machining and seeding of stem cells.

Herein, we demonstrate that spin‐coating, a technique commonly used to obtain thin films with well‐controlled thicknesses, can be adapted to fabricate scaffolds with mineral gradients mimicking that of the native insertion. Taking the future clinical translation into consideration, we decided to focus on HAp (a widely used inorganic biomaterial due to its high mechanical strength and superior osteoconductivity) and PCL (a synthetic polymer known for its excellent biocompatibility and in vivo biodegradability).^[^ ^10^ ^]^ Both materials have been approved by regulatory authorities, including the United States Food and Drug Administration, for use in human subjects.^[^ ^11^ ^]^ Specifically, HAp/PCL suspensions with decreasing concentrations of HAp nanoparticles were sequentially spin‐coated on a silicon wafer (**Figure** **1**). During spin‐coating, most of the solvent evaporated, resulting in a uniform PCL thin film with evenly distributed HAp nanoparticles. As the HAp/PCL suspension of the subsequent layer was deposited, the freshly introduced solvent helped anneal the adjacent two layers, and at the same time, gave rise to a smooth interface between them. As a major advantage over prior methods, the high reproducibility of spin‐coating ensured both layer‐to‐layer and batch‐to‐batch consistency. In addition, the steepness of the gradient and the length scale of the mineral‐graded zone could be conveniently tuned by adjusting the number of layers with specific HAp/PCL concentrations, enabling a customizable scaffold design. In principle, this method can be readily extended to other combinations of polymers and inorganic nanoparticles. For the current study, after obtaining a large spin‐coated film, multiple smaller scaffolds with funnel‐shaped microchannels were laser‐machined. The morphology of the microchannels was also optimized by adjusting the laser parameters. Human‐derived mesenchymal stem cells (hMSCs) were then seeded into the microchannels and induced to take on distinctive differentiation pathways depending on the local HAp contents they were exposed. When implanted between the tendon and bone, such a scaffold could serve as a template for the regeneration of a robust tendon enthesis, augmenting the repair of this interfacial tissue that is prone to failure. In the current study, we focus on demonstrating the effect of the graded scaffolds in vitro. Future animal experiments with clinically relevant models will be carried out to test their efficacy in rotator‐cuff repair.

## 2 Results and Discussion

### 2.1 Characterization of the Gradient in HAp Content of the Graded Scaffolds

We began the fabrication process by choosing the right combinations of materials. In the form of nanoparticles, HAp could be evenly distributed in a PCL solution to form a suspension with a relatively high viscosity. In principle, the result of spin‐coating depends heavily on the angular velocity or spin speed and the physical properties of the material to be spun. When the weight percentage of PCL in the polymer solutions was increased from 6 to 8 and 10 wt.%, the thickness of the resulting film after 20 layers of spin‐coating increased from 24.38 ± 0.74 µm to 46.88 ± 0.83 µm and 103.38 ± 1.06 µm, respectively (N = 10). To provide enough mechanical strength and adequate z‐depth for the infiltration of cells into the scaffold, the 10 wt.% PCL solution was used henceforth as the continuous phase. After adding in HAp nanoparticles, energy‐dispersive X‐ray spectroscopy (EDX) mapping was used to verify that HAp was uniformly distributed within the film fabricated by spin‐coating multiple layers of a suspension of HAp/PCL at a fixed weight ratio (Figure S1, Supporting Information). When a single layer of 50/50 (wt./wt.) HAp/PCL suspension was spin‐coated, a thin film with a thickness of 5.67 ± 1.24 µm was obtained (N = 15).

**Figure 2:**
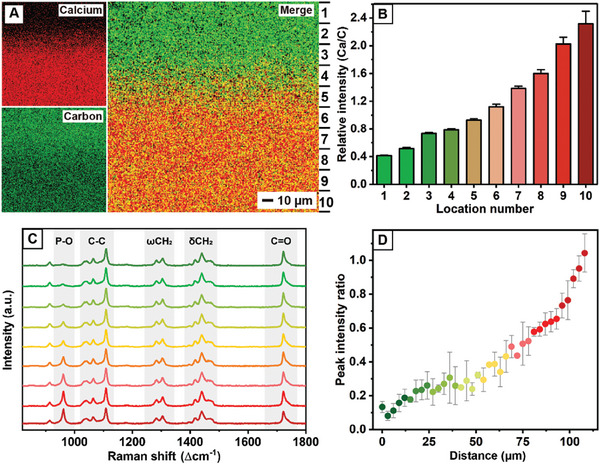
Graded distribution of HAp nanoparticles along the vertical direction of the scaffold fabricated using layer‐by‐layer spin‐coating. A) EDX mapping of the graded cross‐section, with carbon and calcium shown in green and red, respectively. B) Quantification of calcium/carbon ratio along the vertical direction of the scaffold based on the EDX data (N = 10). C) Representative Raman spectra were recorded from different regions of the cross‐section along the vertical direction of the scaffold. D) The plot of the ratio between the intensities of the peaks at 960 cm^−1^ (P─O stretch) and 1724 cm^−1^ (C=O stretch), which correspond to HAp and PCL, respectively (N = 3). The data sets in panel (B) and (D) were presented as mean ± standard deviation.

Next, we examined the gradient of HAp along the vertical direction of the scaffold through EDX mapping. To resolve the gradient through its depth, the scaffold was cryo‐sectioned into slices 50 µm thick. As shown by the EDX mapping in **Figure** **2**A, the calcium content (red color) was concentrated at the bottom of the scaffold and gradually decreased toward the top. A semi‐quantitative analysis of the relative intensity of calcium demonstrates that the HAp concentration changed monotonically along the vertical direction of the scaffold (Figure 2B). The HAp gradient can be attributed to the variations of HAp concentration in the suspensions used for the layer‐by‐layer spin‐coating process. The steepness of the gradient and the length scale of the mineral‐graded zone could both be readily tuned by adjusting the step size of the change in HAp concentration between two consecutive layers and the number of layers spin‐coated at each HAp concentration (Figure S2, Supporting Information). In addition, three gradient patterns were created by spin‐coating PCL solutions containing varying concentrations of a fluorescent dye (Figure S3, Supporting Information). The gradient formed closely followed the expected trend. This result further demonstrates the tunability and versatility of the spin‐coating method.

Raman spectroscopy data corroborated the EDX mapping results, demonstrating a monotonic change in HAp concentration through the depth of the scaffold. As shown in Figure 2C, the Raman shift intensity of the P‐O stretches at 960 cm^−1^ increased along the vertical direction, indicating a rise in HAp concentration. Simultaneously, the peaks corresponding to PCL ≈1031–1109 cm^−1^ (C─C stretch), 1281−1306 cm^−1^ (CH_2_ bend), 1418−1474 cm^−1^ (CH_2_ twist), and 1724 cm^−1^ (C═O stretch) either remained constant or decreased slightly in intensity. As shown in Figure 2D, the relative concentration of HAp at each location and the ratio between the intensities of the P─O stretch (960 cm^−1^) and C═O stretch (1724 cm^−1^) peaks were correlated. Taken together, these results clearly demonstrate the successful fabrication of an HAp‐graded scaffold using the layer‐by‐layer spin‐coating method.

### 2.2 Optimization of the Laser‐Machined Microchannels for Cell Seeding

We next focused on transforming the HAp‐graded polymer film into a functional scaffold for tendon‐to‐bone repair. To this end, we used a femtosecond laser to drill a 10 × 10 array of funnel‐shaped microchannels through the film to allow stem cell infiltration and then graded differentiation. In the native enthesis, the mineralized and unmineralized tissues adopt an interdigitation geometry. Biomechanical modeling studies revealed that this structure is critical for optimizing the balance between strength and toughness, ultimately alleviating stress at the interface to produce a tough attachment.^[^ ^12^ ^]^ To achieve this feature in our scaffold, the microchannels were designed to take on a funnel shape, with a larger opening on the side with lower HAp content and a smaller one on the HAp‐enriched side. Our previous study showed that Young's modulus of an HAp/polymer composite is closely correlated to the concentration or density of HAp present in the material.^[^ ^13^ ^]^ Since the HAp distribution varies along the depth of the scaffold, the cells adhering to the microchannel side walls are exposed to a compositional gradient and the associated variation in mechanical properties. It is well‐established that cell behavior, particularly stem cell differentiation, is strongly affected by the matrix property, such as the stiffness of the substrate to which they adhere.^[^ ^14^ ^]^ Maximizing the population of cells attaching to the side walls of the microchannels would be essential for potentially generating an osteogenic to chondrogenic cell phenotype gradient to facilitate enthesis repair. We hypothesize that the morphology of the microchannels, specifically the slope angle, will significantly impact the efficacy of stem cell seeding. By tuning the fabrication parameters, including the laser power, movement speed, and repeating cycles, we could consistently produce microchannels with the desired slope and optimize the scaffold for stem cell attachment, proliferation, and differentiation.

**Figure 3:**
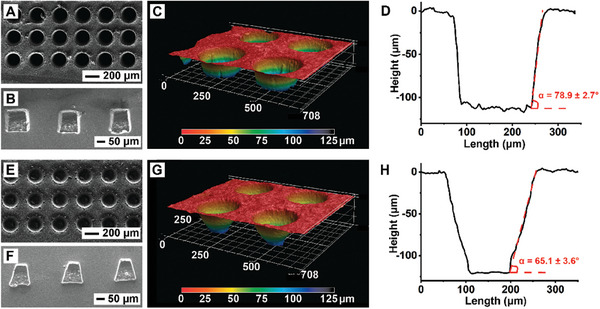
Characterizations of the funnel‐shaped microchannels with different slope angles. A) Top and B) cross‐sectional SEM images and C) 3D and D) cross‐sectional profile of the microchannels with a slope angle of ≈80°. E) Top and F) cross‐sectional SEM images and G) 3D and H) cross‐sectional profile of the microchannels with a slope angle of ≈65°. An optical surface profiler (Keyence VK‐X3000) in a focus variation mode and its associated analyzer software was used to obtain the 3D and cross‐sectional profiles shown in panels (C), (D), (G), and (F).

As shown by the scanning electron microscope (SEM) images of the cryo‐sectioned scaffold and the 3D profile in **Figure** **3**, the top opening size (diameter) and the center‐to‐center separation distance between adjacent microchannels were fixed at 221.0 ± 5.0 µm and 146.7 ± 14.52 µm, respectively (N = 24). The top opening size was chosen such that the hMSCs (15–30 µm in size^[^ ^15^ ^]^) could easily infiltrate into the microchannels and there would be sufficient room for bundles of collagen fibrils (80–320 µm in diameter^[^ ^16^ ^]^) to develop and reconstruct anatomical characteristic of the tendon. During the laser machining process, we were able to establish a correlation between the energy density of the laser and the microchannel profile. The amount of material removed was positively correlated with the laser power and repeating cycles, and negatively correlated with the laser scanning speed. To drill each microchannel, the laser followed the path of concentric circles. By increasing the number of repeating cycles from the peripheral to the center, we consistently obtained microchannels with a tapered cross‐sectional profile resembling that of a funnel. On top of this, the slope angle was reduced by decreasing the laser power and increasing the scanning speed. Using this approach, we produced scaffolds with the slope of the microchannels set to either 78.9 ± 2.7° or 65.1 ± 3.6° (N = 24) (Figure 3D,H). After fabrication, an optical surface profiler was used to measure the profile of the microchannels non‐invasively. Following this, we proceeded with in vitro experiments to study how the different scaffold geometry would affect cellular behavior, particularly the attachment during the seeding stage.

### 2.3 Cell Growth in the Scaffolds with Different Slopes of the Microchannels

Mesenchymal stem cells (MSCs) have demonstrated multipotent differentiation capabilities.^[^ ^17^ ^]^ Due to their abundance and easily accessible locations in the human body,^[^ ^18^ ^]^ MSCs have been increasingly utilized in tissue engineering studies, with many successes in enthesis and tendon repairs.^[^ ^19^ ^]^ By incorporating human‐derived MSCs into the HAp‐graded scaffold, we evaluated its capability as an instructive template in driving the formation of a cell differentiation gradient. Furthermore, from a translational standpoint, this hMSC‐seeded biomimetic scaffold not only provides a source of stem cells for the regeneration of the transitional tissue but also potentially initiates the reconstruction of the proper extracellular matrix and mineralization essential for the tendon enthesis.

We first analyzed the biocompatibility of the HAp‐graded scaffold using live/dead staining. The hMSCs showed high viability, with negligible death observed after 24 h of culture (Figure S4, Supporting Information). This result indicates that the HAp/PCL scaffolds have excellent biocompatibility.

**Figure 4:**
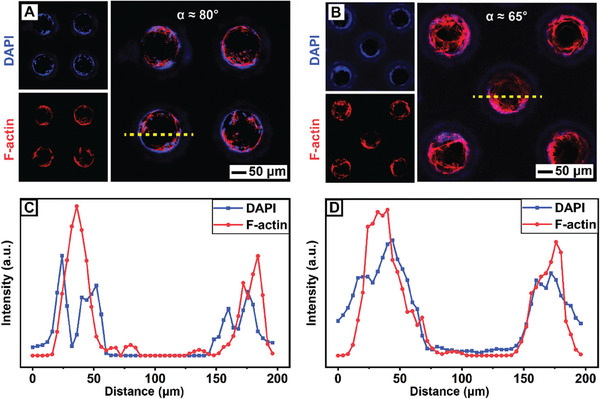
The distribution of cells in the HAp‐graded scaffolds with two sets of slope angles. A,B) Frontal plane orthogonal projection of the z‐stack fluorescence images of the scaffold bearing a slope angle of (A) ≈80° and B) ≈65°, respectively, after 24 h of culture. C,D) Average fluorescence intensity distribution across the microchannel, following the path marked by the yellow dotted line in panels (A) and (B), with a sample size of 5 (N = 5). In (A) and (B), the cell nucleus was stained blue with DAPI, and the F‐actin was stained red. The orthogonal projection was done using the weighted average method in Zen (blue) software by Zeiss. Each microchannel is considered an independent technical replicate.

Proper seeding of hMSCs sets the foundation for subsequent proliferation and graded differentiation. To optimize the scaffold structure for this step, we seeded hMSCs onto scaffolds with different slope angles of ≈80° and 65°, respectively, and analyzed their distribution inside the microchannels through immunostaining. After 24 h of culture, the cell nuclei and F‐actin were stained with 4′,6‐diamidino‐2 phenylindole (DAPI) and Phalloidin‐549, respectively. As shown by the frontal plane projection confocal microscopy micrographs (**Figure** **4**A,B), most cells were located within the microchannels and attached to the walls. Comparing the scaffolds with different slope angles, the group with a gentler slope (α ≈ 65°) showed a stronger F‐actin signal, indicating a larger population of cells successfully attached to the scaffold after seeding. In addition, we semi‐quantitatively evaluated the DAPI and Phalloidin‐549 signal across the microchannel (Figure 4C,D). The signals from the scaffold bearing a slope angle of ≈65° extended more toward the center of the channel, closely following the profile of the funnel‐shaped morphology. This result further implied that good attachment of cells was achieved with a smaller slope angle. SEM images of the hMSC‐seeded scaffold after 24 h of culture supported this observation (Figure S5, Supporting Information). Higher magnification images of the scaffold bearing a slope angle of ≈65° revealed that the stem cells exhibited a spread‐out morphology after adhering to the walls of the channel. This phenomenon was not observed on the scaffold with a larger slope angle. The influence of the different slope angles is sustained until a later time point in the cell culture. After 14 days of culture, the cytoskeleton of the hMSCs almost filled up the entire center of the microchannels for the group bearing a slope angle of ≈65°, while those in the ≈80° group mostly populate the periphery of the microchannels (Figure S6, Supporting Information). Based on these observations, we concluded that the scaffolds with microchannels featuring a slope angle of ≈65° give rise to better cellular attachment and spread along the side walls with HAp gradient. Therefore, we continued with scaffolds featuring this morphology in the subsequent in vitro studies.

### 2.4 A Gradient in Cell‐Graded Differentiation in the Graded Scaffolds

Recreating the complex tendon‐to‐bone attachment gradient in composition, structure, and cell phenotype is essential to augment repair. A gradient in cell phenotype exists at the natural tendon enthesis: tendon fibroblasts populate the tendon, chondrocyte‐like cells populate the enthesis fibrocartilage, and osteoblasts/osteocytes populate the bone.^[^ ^1c^ ^]^ Our previous studies indicated that a mineral gradient can drive local cellular differentiation, creating graded osteogenesis along the mineral gradient.^[^ ^8^, ^9^ ^]^ In the current study, we further demonstrated that the HAp‐graded scaffold could induce a gradual change in cell phenotype from osteoblasts to mineralized chondrocytes. To this end, we assessed both chondrogenesis and osteogenesis of the hMSCs in the scaffolds with immunostaining after 14 days of proliferation and differentiation.

Lineage‐specific markers were chosen to identify cells along distinctive differentiation pathways. Specifically, type X collagen (Collagen X) was selected as a differentiation marker for hypertrophic (i.e., mineralizing) chondrocytes, consistent with the expression patterns by mineralized chondrocytes in the tendon enthesis.^[^ ^20^ ^]^ Osteopontin (OPN), a widely recognized osteogenic marker, was used to identify cells undergoing osteogenesis.^[^ ^21^ ^]^ Secondary antibodies bearing red (Alexa Fluor 594) and green (Alexa Fluor 488) fluorescence were used to aid the observation of Collagen X and OPN, respectively.

**Figure 5:**
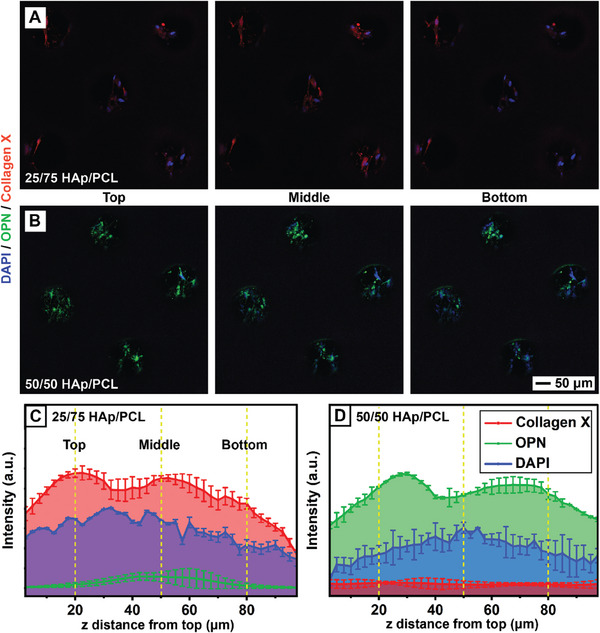
Chondrogenic and osteogenic differentiation of hMSCs in the control group of scaffolds with uniform HAp distribution. A,B) Top view confocal micrographs at different z planes of the scaffolds fabricated with A) 25/75 HAp/PCL and B) 50/50 HAp/PCL, respectively. C,D) Quantitative analysis of Collagen X, OPN, and DAPI expression along the vertical direction of the scaffold corresponding to the images in (A) and (B), respectively. Data was presented as mean ± standard deviation. Average intensities obtained from six microchannels (N = 6) are presented.

When the immunocytochemical study was conducted on control groups of scaffolds with a uniform distribution of HAp through the depth of the microchannels, no cell phenotype gradient was observed. As shown in the confocal micrograph images in **Figure** **5**A, the hMSCs in the scaffolds fabricated by spin‐coating 20 layers of 25/75 (wt./wt.) HAp/PCL suspension showed consistently expressed Collagen X from the top to the bottom of the microchannels, while those in the scaffolds fabricated with 50/50 (wt./wt.) HAp/PCL suspension predominantly expressed OPN in Figure 5B. A semi‐quantitative analysis of the fluorescence intensities of Collagen X, OPN, and cell nuclei (DAPI) indicates that the expression of OPN and Collagen X were only on the level of noises in the 25/75 HAp/PCL and 50/50 HAp/PCL scaffolds, respectively (Figure 5C,D).

**Figure 6:**
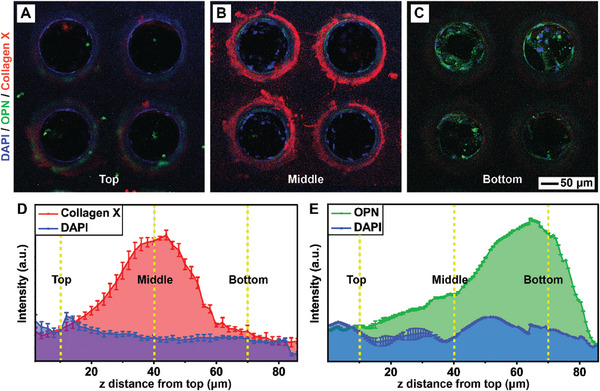
Chondrogenic and osteogenic differentiation of hMSCs in the HAp‐graded scaffolds. A–C) Top view confocal micrographs at different z planes of the scaffold with a slope angle of ≈65° after 14 days of culture. D,E) Quantitative analysis of (D) Collagen X and (E) OPN expression along the vertical direction of the scaffold corresponding to the images in (A–C). Data was presented as mean ± standard deviation. Average intensities obtained from four microchannels (N = 4) are presented.

In comparison, as shown in the confocal fluorescence micrographs in **Figure** **6**A–C, on day 14, the expression of Collagen X was most intense in the middle section of the scaffold, while that of OPN was the greatest at the bottom. A semi‐quantitative analysis of the fluorescence intensities of Collagen X, OPN, and DAPI with respect to the vertical distance from the top surface of the scaffold further confirmed the graded expression (Figure 6D,E). Like in the control group, the intensity of DAPI (blue color) did not vary significantly along the depth of the microchannel despite slight fluctuations. Its consistent presence through the measured distance indicates a relatively uniform distribution of hMSCs along the side walls. In comparison, neither Collagen X nor OPN is distributed evenly through the vertical direction. There was a peak in the Collagen X intensity curve ≈30–50 µm from the top, whereas the intensity of OPN was higher toward the bottom of the scaffold. This data suggests that the hMSCs located in the middle of the scaffold exhibited a stronger chondrogenic trend, and those at the bottom were more likely committed toward an osteogenic path. These results imply that the scaffold supports an osteogenic to chondrogenic differentiation gradient through the depth of the pores.

As shown in Figure 2, the scaffold in the current study, fabricated using layer‐by‐layer spin‐coating, exhibited a monotonic decrease in HAp concentration from the bottom to the top, mimicking the mineral composition of the native enthesis. Prior studies have shown that substrate stiffness alone, without additional exogenous stimuli, is a key factor in driving the osteoblastic and chondrogenic differentiations, with a less stiff substrate promoting a stronger chondrogenic response than an osteogenic response.^[^ ^22^ ^]^ As discussed above in Section 2.2, our group has previously shown that increasing the concentration of HAp in an HAp/polymer composite would lead to an increase in the Young's Modulus, or essentially the stiffness, of the material. Driven by a combination of spatial gradient in biochemical (i.e., HAp nanoparticles) and mechanical (i.e., substrate stiffness) cues, a cell phenotype gradient was formed over different zones along the depth of the channels.^[^ ^8^, ^23^ ^]^ The comparison to the control group scaffolds with a uniform HAp distribution further supports our argument that the gradient of HAp is vital in providing these cues. The observed cell phenotype gradient matched that of the native tendon enthesis, a feature critical for augmenting functional recovery after tendon‐to‐bone repair.^[^ ^8^ ^]^ The osteogenic to chondrogenic transition may help induce the regeneration of the enthesis extracellular matrix by facilitating the production of collagen fibrils and further enhancing the gradual mineralization of the connective tissue. Taken together, the scaffold holds promise for augmenting tendon‐to‐bone repair.

## 3 Conclusion

Using the characteristics of the healthy tendon enthesis as design criteria, we have developed a facile method for fabricating functionally graded scaffolds with well‐controlled HAp gradients using the spin‐coating method. The steepness and length scale of the HAp gradient, as well as the overall thickness of the scaffold, can be easily tuned to match those of the native enthesis. More importantly, we demonstrated that the scaffold could be seeded with stem cells, which were then prompted to form a cell phenotype gradient consistent with the native enthesis. By exposing hMSCs to the HAp gradient through the depth of the scaffold, we achieved a controllable gradient in osteogenic‐to‐chondrogenic cell phenotype. The scaffold may facilitate in vivo reconstruction of the tendon enthesis by producing an extracellular matrix unique to this transition zone. The simple, yet reliable, fabrication method allows for batch production with high yield, enabling it to be readily applied in clinically relevant translational studies. Moreover, the results will be broadly applicable to connective tissue‐to‐bone repair in other locations, including anterior cruciate ligament reconstruction and meniscal repair.

## 4 Experimental Section

### Chemicals and Materials

PCL (Mn ≈80000), 1,4‐dioxane, paraformaldehyde, poly‐l‐lysine, Triton X‐100, rhodamine B (RhB), and bovine serum albumin (BSA) were all purchased from Sigma‐Aldrich. Fetal bovine serum (FBS), 𝛼‐minimum essential medium, and antibiotic‐antimycotic were obtained from Gibco. Phosphate‐buffered saline (PBS, pH 7.4), Calcein‐AM, ethidium homodimer‐1 (EthD‐1), and normal goat serum were obtained from Thermo Fisher. The optimal cutting temperature (OCT) medium was obtained from Sakura Finetek USA Inc. The HAp nanoparticles (<200 nm in size) used in this work were ordered from Sigma‐Aldrich and ground with a mortar and pestle before use. The water used in all experiments was obtained from a Millipore ultrapure water system (Billerica, MA).

### Fabrication of PCL Film with HAp Gradient

The PCL film with a vertical gradient in HAp was fabricated using a layer‐by‐layer spin‐coating method. Specifically, different amounts of grounded HAp powder were mixed with 10 wt.% of PCL in 1,4‐dioxane under vortexing and ultrasonication. Two layers were spin‐coated onto a 4‐inch wafer from each suspension of 50/50, 45/55, 40/60, 35/65, 30/70, 25/75, 20/80, 15/85, 10/90, and 5/95 (wt./wt.) HAp/PCL using a spin‐coater (Chemat Technology, KW‐4A). Briefly, 1.5 mL of the HAp/PCL suspension was deposited on the wafer for each layer, and the spinner was set to 1.46 krpm and ran for 63 s. After spin‐coating of all the 20 layers, the film was placed in the fume hood overnight for the solvent to evaporate. It was then released from the wafer for further processing. Films with different HAp distribution can be readily fabricated by changing the set of suspensions spin‐coated. Films using 6 and 8 wt.% PCL were also spin‐coated in the same manner. The thickness of the film was measured with a REXBETI digital micrometer with 1 µm resolution. Due to its resolution limit, the thickness measurement was taken after 20 layers were spin‐coated for each polymer concentration.

### Fabrication of PCL Film with Rhodamine B Gradient

The PCL film with a vertical gradient in RhB was fabricated similarly to that described above. To create different gradient patterns, the following three sets of spin‐coating were performed. For pattern A, three layers were spin‐coated onto a 4‐inch wafer from each solution with 0.7, 0.6, 0.5, 0.4, 0.3, 0.2, 0.1, 0.05 mg mL^−1^ RhB; for pattern B, two layers were spin‐coated onto a 4‐inch wafer from each solution with 0.5, 0.45, 0.4, 0.35, 0.3, 0.25, 0.2, 0.15, 0.1, 0.05 mg mL^−1^ RhB; for pattern C, four layers from solutions with 0.3 and 0.05 mg mL^−1^ RhB were spin‐coated at the beginning and the end, and three layers from each solution with 0.25, 0.2, 0.15, and 0.1 mg mL^−1^ RhB were spin‐coated in between. The films were released from the wafer after drying and the distribution of RhB along the depth of the film was imaged using a confocal fluorescence microscope (Zeiss LSM 700).

### Laser Machining to Create Channels with Tapered Angles

A femtosecond laser (OPTEC WS‐Flex) with a spot size of 20 µm was utilized to drill a 10 × 10 array of microchannels for cell infiltration. A CAD file with the designed pattern, for example, an array of concentric circles, was prepared using AutoCAD 2022 and uploaded to the laser controller to generate a path file. A slab of the HAp‐graded film was fixed on a glass panel attached to the moving platform of the laser machine. The laser was set to 4 kHz, 39 W power, and 80 mm s^−1^ speed for fabricating the microchannels bearing a slope angle of ≈65°, and 4 kHz, 18 W power, and 100 mm s^−1^ speed for those bearing a slope angle of ≈80°. After the microchannels were created, the laser was set to run five passes at 4 kHz, 60 W power, and 10 mm s^−1^ speed to punch out small circular disks (D = 5 mm) with the array of microchannels situated in the center.

### EDX and Raman Imaging of the Gradient of HAp

The scaffold was vertically cryo‐sectioned into 50 µm slices for the characterization of HAp distribution along the vertical direction of the scaffold. Specifically, the scaffold was treated with a plasma cleaner (Plasma Etch PE50) for 2 min to make the surface hydrophilic. It was then embedded in OCT in a cryomold and frozen at −80 °C for 1 h before cryo‐sectioning. The OCT‐embedded sample was loaded on a cryostat (Thermo Scientific CryoStar NX70) and sectioned perpendicular to the base at a thickness of 50 µm. The sectioned slices were collected on a glass slide with a double‐sided adhesive tape. The OCT was removed by rinsing with H_2_O three times, followed by drying in a fume hood for 1 h. The cryo‐sectioned samples were subsequently examined with EDX (Hitachi SU8230) at 20 kV and 30 µA. The calcium and carbon distributions were semi‐quantitatively analyzed using ImageJ software. The distribution of HAp inside the scaffold was measured using a Renishaw inVia Raman spectrometer (Wottonunder‐Edge) coupled with a Leica optical microscopy (Leica Camera Wetzlar). Raman data was collected along a line of 108 µm with a step size of 3 µm. For each data point, the spectrum was collected with a 20× objective lens, a 488 nm laser with 50 mW power, 2400 lines per mm grating, and a collection time of 0.3 s. Signal‐to‐baseline intensity mappings for the Raman shift peaks at 960 and 1724 cm^−1^ were constructed using the WiRE 5.2 software. The HAp concentration is correlated with the intensity ratio between these two peaks.

### Structural Characterization of the Scaffold

SEM (Hitachi SU8230) was used to visualize the morphology of the microchannels. An intact scaffold or a glass slide with the cryo‐sectioned scaffolds was sputtered with 2–3 nm Au using a Hummer 6 Sputtering System for 55 s at 30 mA. The electron beam was set at 5 kV and 10 µA for imaging. To further quantify the profile of the funnel‐shaped microchannels, the scaffolds were characterized using an optical surface profiler (Keyence VK‐X3000) in a focus variation mode.

### Isolation, Culture, and Seeding of hMSCs

Human‐derived MSCs were previously obtained from a commercial source (Lonza, Basel, Switzerland) and were recovered from cryopreservation. After being recovered from cryopreservation, the cells were cultured until the third generation in an α‐MEM medium supplemented with 10% FBS and 1% antibiotics (containing penicillin and streptomycin) prior to being seeded.^[^ ^24^ ^]^ The scaffolds were treated with a plasma cleaner (Plasma Etch PE50) for 2 min to increase their surface hydrophilicity and facilitate cell adhesion. They were then sterilized in 70% ethanol overnight, rinsed with PBS three times, and immersed in an aqueous solution of poly‐l‐lysine (1 mg mL^−1^) and shaken at 50 rpm overnight, followed by immersing in an α‐MEM medium supplemented with 10% FBS overnight. The size of each slab of the scaffold was designed to fit tightly at the bottom of a 96‐well culture plate (Corning) to allow for maximum seeding efficiency. The hMSCs were seeded at a density of 5 × 10^5^ cells mL^−1^ by pipetting a suspension of cells directly to the top of the microchannels. The culture plate with the scaffolds was shaken on a 3D shaker platform (Corning GyroTwister S1000‐40) at 30 rpm for 30 min, followed by incubation at 37 °C in a humidified chamber containing 5% CO_2_ overnight to allow the cells to sediment and attach to the scaffold. The culture medium was changed every other day.

### Live/Dead Assay for Scaffold Biocompatibility

Calcein‐AM and EthD‐1 were used to stain the live and dead cells, respectively. After 24 h of culture, cells were incubated with serum‐free α‐MEM containing 5 mm calcein‐AM and 4 µm EthD‐1 at 37 °C for 30 min. After washing with PBS three times, the samples were observed under a confocal fluorescence microscope (Zeiss LSM 900).

### Cell Distribution in the Scaffolds

Confocal fluorescence micrographs and SEM images were used to evaluate the quality of hMSC seeding and attachment to the walls of the microchannels. After incubation for 24 h and 14 days, the scaffolds were retrieved for the staining of F‐actin and nuclei. Briefly, they were fixed with 4% paraformaldehyde for 20 min, permeabilized in 0.1% Triton X‐100 for 10 min, and then incubated in 1% BSA for 30 min to avoid nonspecific binding. Afterward, the scaffolds were incubated with Alexa Fluor 594‐conjugated phalloidin (Thermo Scientific) at a dilution of 1:200 for 30 min at room temperature to stain the F‐actin, followed by incubation with 1 µg mL^−1^ DAPI (Thermo Scientific) for 10 min to stain the nuclei. After washing with PBS, the scaffolds were imaged under a confocal microscope (Zeiss LSM 900). The SEM images were obtained by first fixing the cell‐seeded scaffolds in 2.5% glutaraldehyde solution for 30 min. To prevent sample distortion due to the removal of water, sequential dehydration was performed by incubating the samples in a series of solutions with increasing concentrations of ethanol in water (30%, 50%, 70%, 80%, 90%, and 100%). Finally, the samples were imaged using SEM (Hitachi SU8230).

### Immunofluorescence Staining

After 14 days of culture, the cell‐seeded scaffolds were fixed with 4% paraformaldehyde for 20 min, permeabilized in 0.1% Triton X‐100 for 5 min, and then incubated in 1% BSA for 30 min. Afterward, the samples were incubated with primary antibodies detecting chondrogenic differentiation marker Type X collagen (Collagen X, mouse origin) and osteogenic differentiation marker osteopontin (OPN, rabbit origin) at 4 °C overnight, respectively. Subsequently, two different secondary antibodies with different conjugated fluorophores were used to differentiate the specific binding of these two markers. Goat antimouse IgG H&L Alexa Fluor 594 (1:1000 dilution in 1% BSA in PBS) was used for Collagen X and Alexa Fluor 488 goat antirabbit IgG (H+L) (1:200 dilution in 0.2% BSA in PBS) was used for OPN. The samples were incubated with the secondary antibodies for 1 h at room temperature in the dark and imaged with a confocal fluorescence microscope (Zeiss LSM 900). The fluorescence intensity through the depth of the scaffold was analyzed with the Zen (blue) software by Zeiss.

### Statistical Analysis

Data are presented as mean ± standard deviation and “N” indicates the number of samples per group. For the immunocytochemical study, each funnel‐shaped microchannel was considered an independent technical replicate.

## Conflict of Interest

The authors declare no conflict of interest.

## Supporting information

Supporting Information

## Data Availability

The data that support the findings of this study are available from the corresponding author upon reasonable request.
