# Supplementary material for: Reliable Fabrication of Mineral‐Graded Scaffolds by Spin‐Coating and Laser Machining for Use in Tendon‐to‐Bone Insertion Repair
Source: Adv Healthc Mater. 2024 Aug 5;13(31):2402531. doi: 10.1002/adhm.202402531 (PMC11650398; doi:10.1002/adhm.202402531)
Supplement: Supplementary file 1 — Supporting Information [file ADHM-13-0-s001.docx]

**Supporting Information**

**Reliable Fabrication of Mineral-Graded Scaffolds by Spin-Coating and Laser Machining for Potential Use in Tendon-to-Bone Insertion Repair**

Yidan Chen,^#^ Min Hao,^#^ Ismael Bousso, Stavros Thomopoulos, and Younan Xia*

Prof. Dr. Y. Xia, Dr. M. Hao

The Wallace H. Coulter Department of Biomedical Engineering

Georgia Institute of Technology and Emory University

Atlanta, GA 30332 (USA)

E-Mail: younan.xia@bme.gatech.edu

Y. Chen

School of Materials Science and Engineering

Georgia Institute of Technology

Atlanta, GA 30332 (USA)

Prof. S. Thomopoulos, I. Bousso

Department of Orthopedic Surgery

Department of Biomedical Engineering

Columbia University

New York, NY 10032 (USA)

Prof. Y. Xia

School of Chemistry and Biochemistry

Georgia Institute of Technology

Atlanta, GA 30332 (USA)

^#^ These two authors contributed equally to this work.


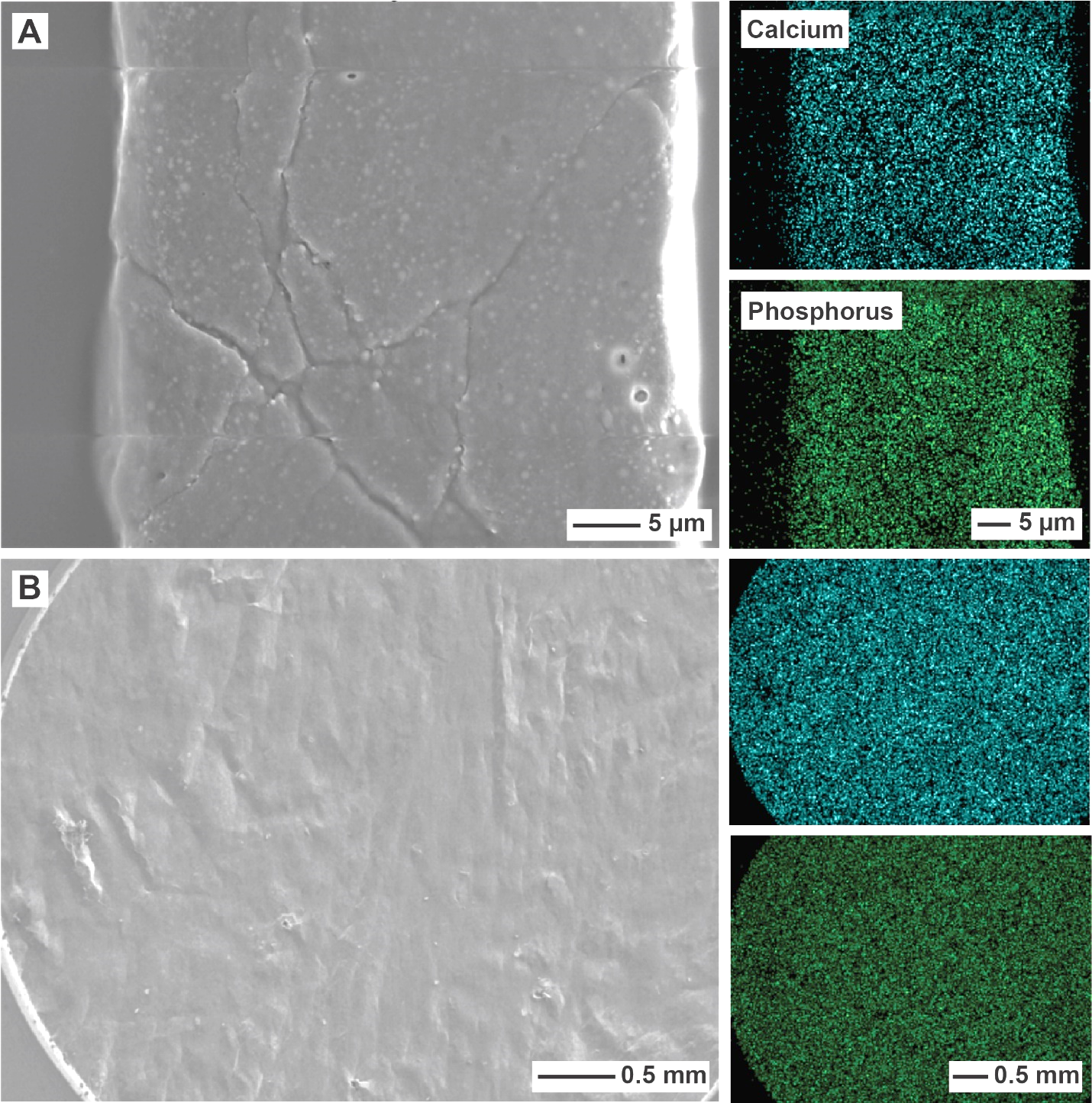


**Figure S1.** Distribution of HAp within a film fabricated by spin-coating 10 layers of a HAp/PCL suspension at a fixed weight ratio (wt./wt.). (A-B) SEM images and EDX mappings of (A) the cross-section and (B) the top of the film, with calcium and phosphorus shown in cyan and green, respectively.


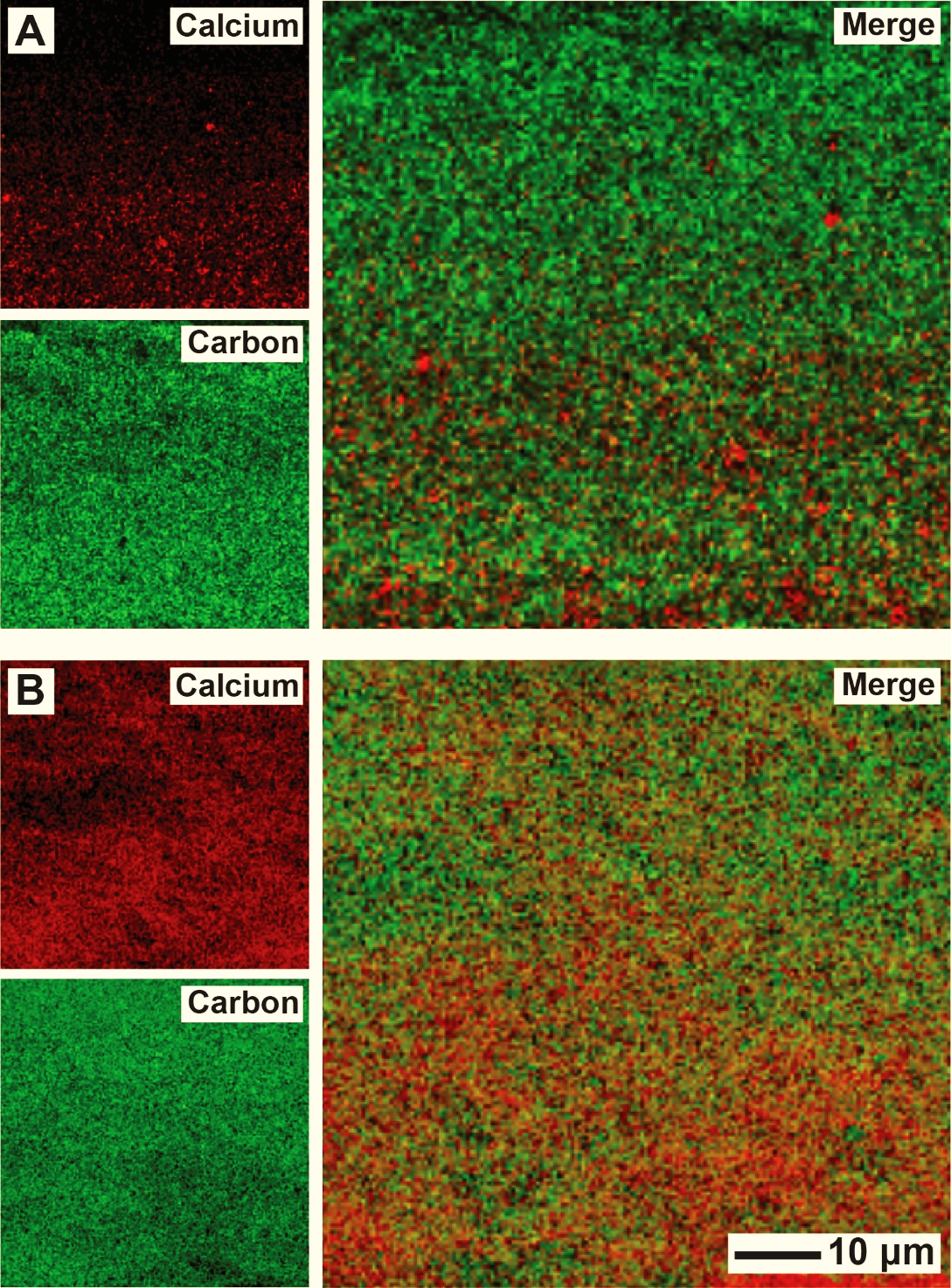


**Figure S2.** Gradient of HAp along the vertical direction of the scaffold. (A-B) EDX mappings of two gradient patterns generated by adjusting the HAp/PCL suspensions used in the spin-coating process, with carbon and calcium shown in green and red, respectively.


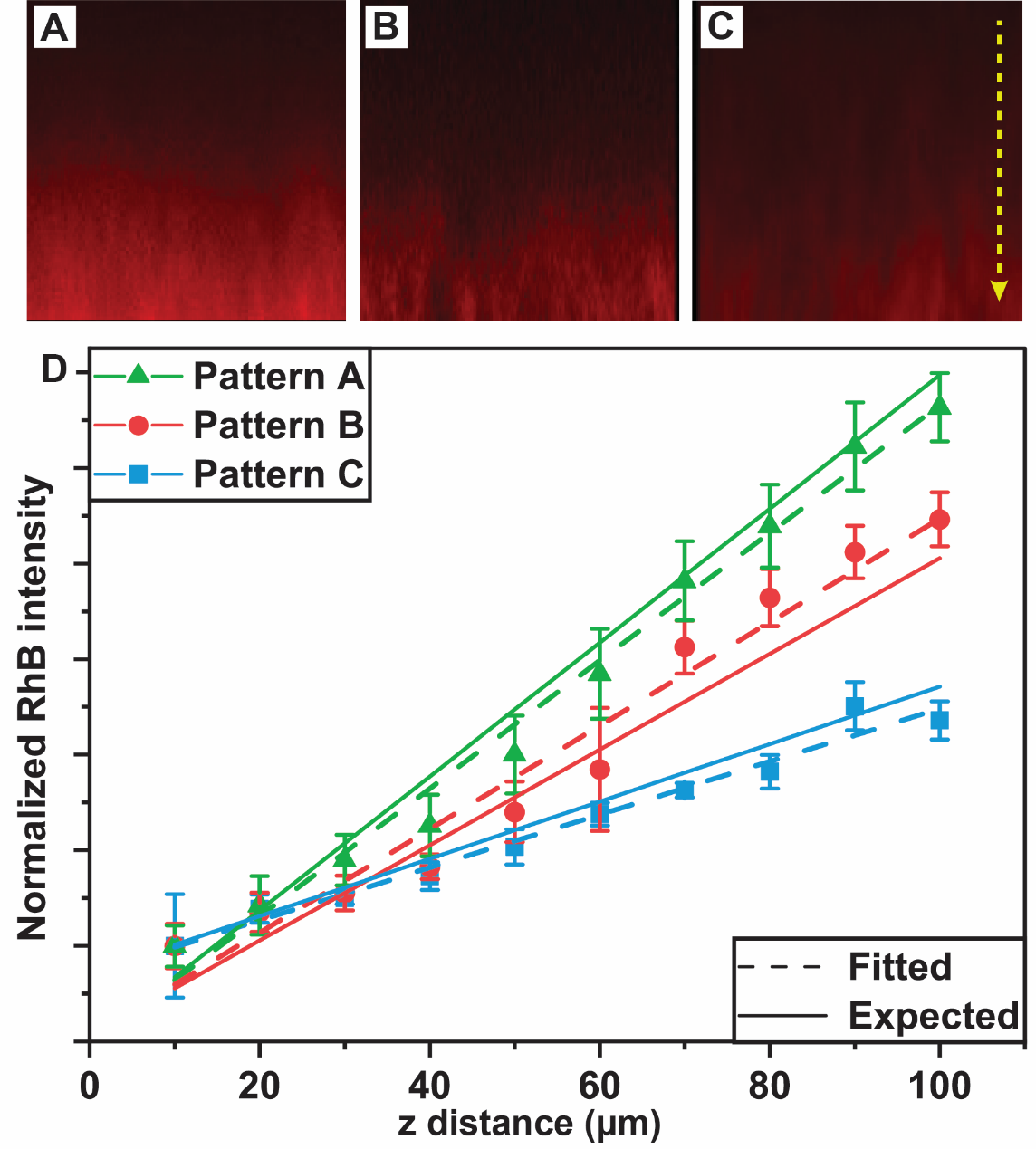


**Figure S3.** Gradient of rhodamine B along the vertical direction of the scaffold. (A-C) Orthogonal views (x/z plane) of the confocal micrographs taken from three samples with different rhodamine B gradients. (D) Quantitative analysis of the fluorescence intensities corresponding to the images in (A-C). Linear curve fitting was performed using Excel on each pattern to obtain the fitted trends. Expected trends were calculated based on the fabrication process, specifically the concentration of RhB in the PCL solutions and the number of layers spin-coated for each concentration. The fitted and expected trends were shown as dotted and solid lines, respectively.


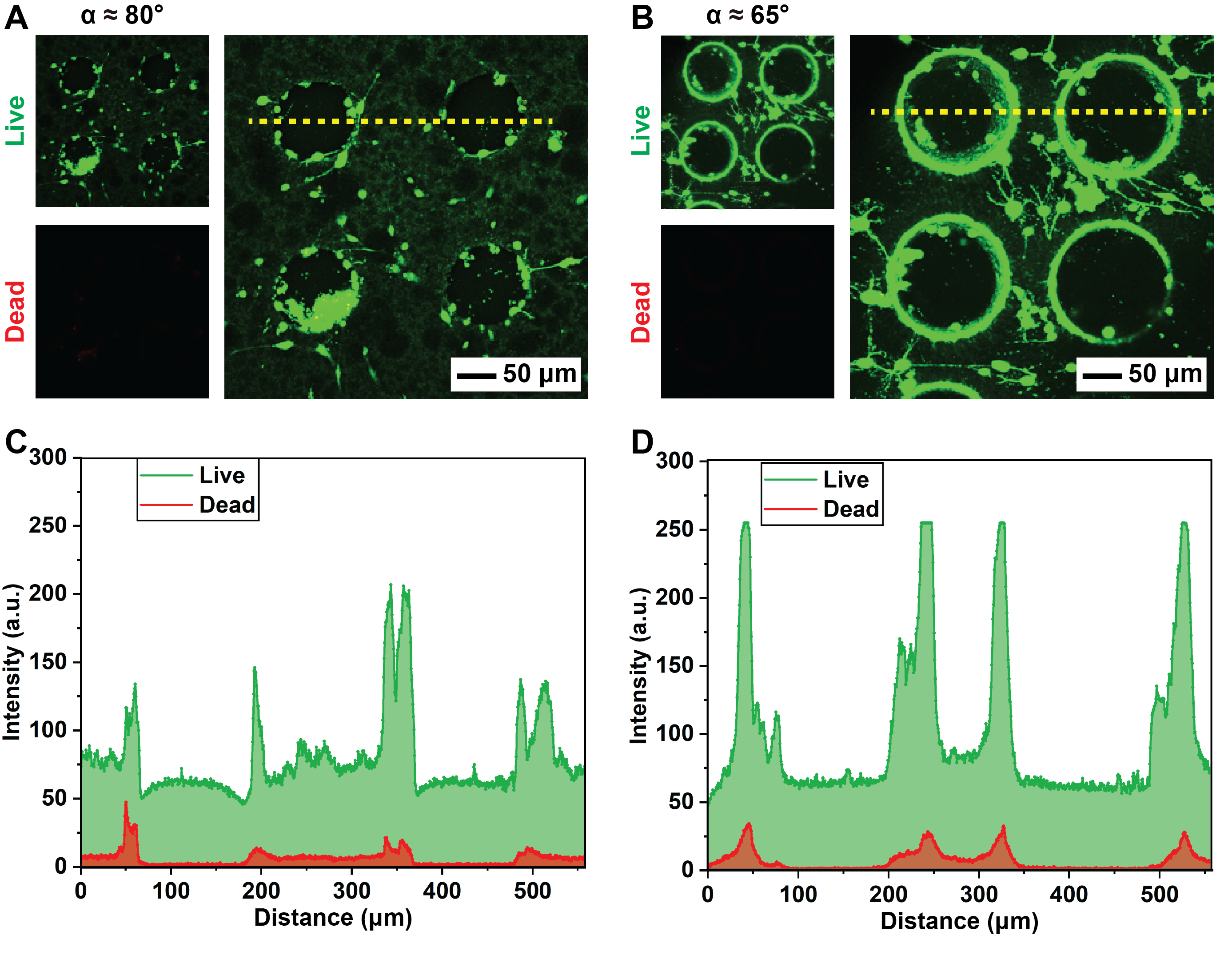


**Figure S4.** Biocompatibility of the HAp-graded scaffolds. Live/dead staining of the hMSCs inside the scaffolds with slope angles of (A) *ca.* 80° and (B) *ca.* 65°, respectively, after 24 h of culture. The live and dead cells were stained green and red, respectively. (C, D) Fluorescence intensity distribution of live/dead staining along the yellow dotted lines in (A) and (B), respectively.


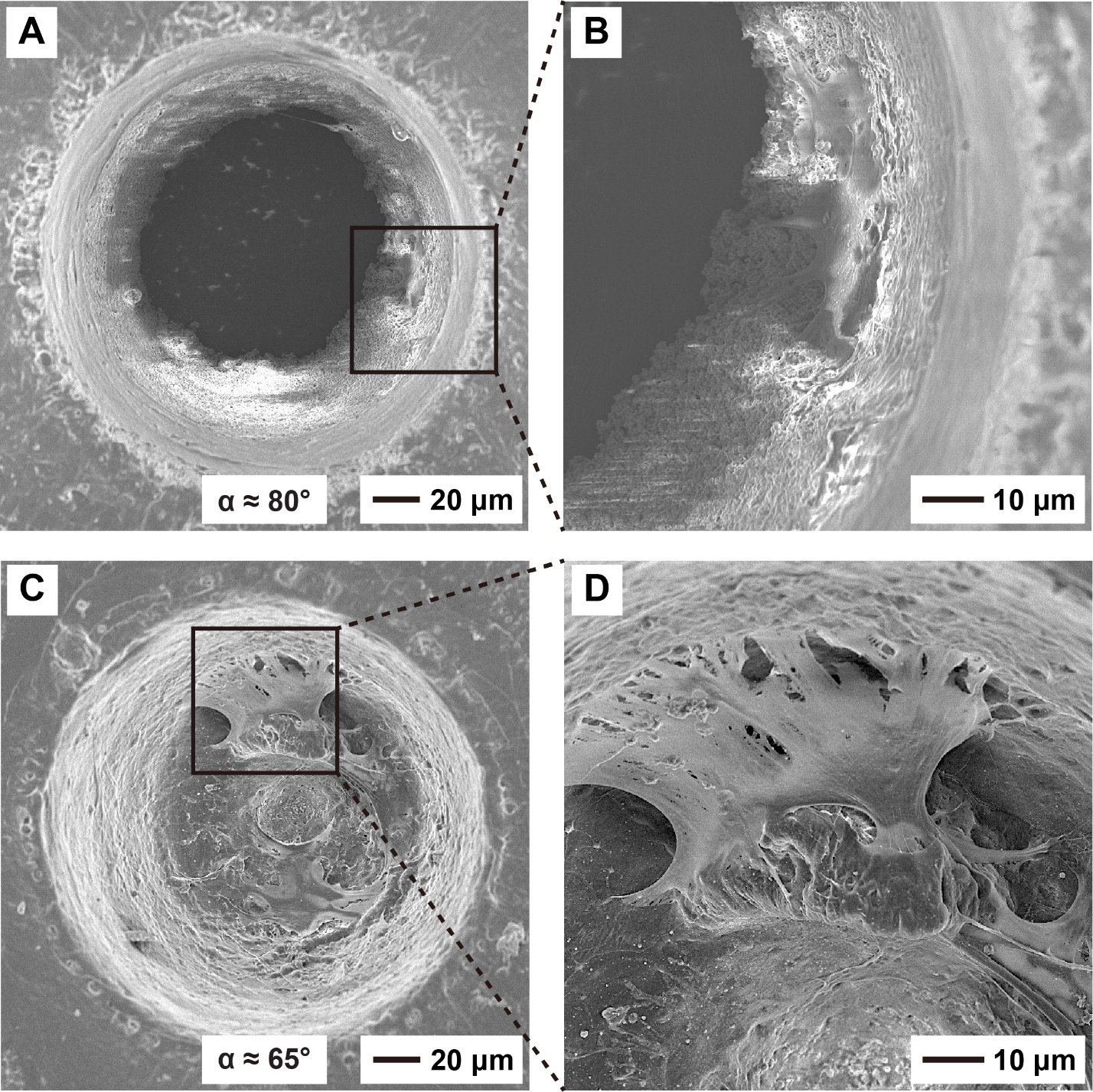


**Figure S5.** SEM images of hMSCs cultured for 24 h with the graded scaffolds bearing slope angles of (A, B) *ca.* 80° and (C, D) *ca.* 65°, respectively.


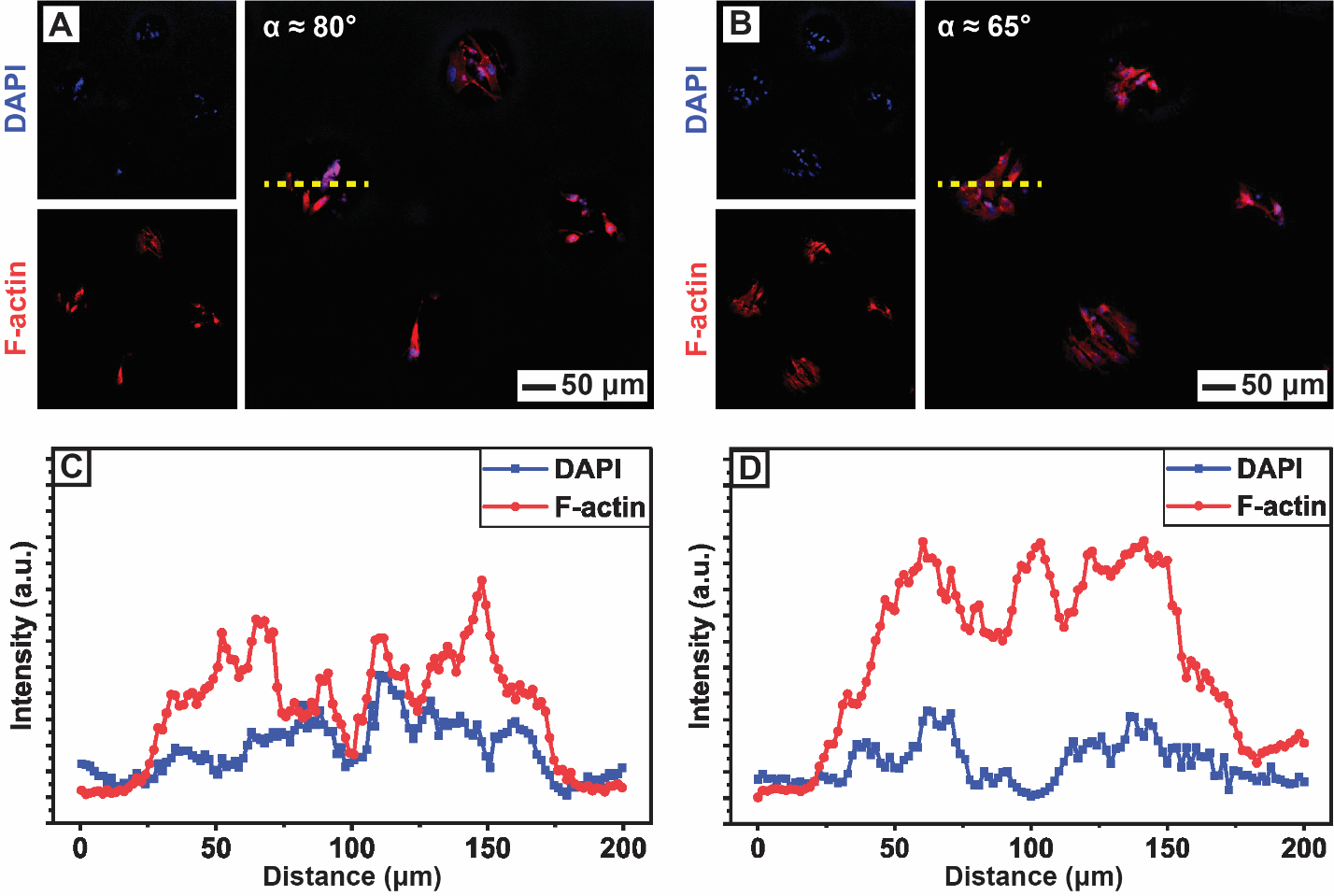


**Figure S6.** The distribution of cells in the HAp-graded scaffolds with two sets of slope angles after 14 days of culture. (A, B) Frontal plane orthogonal projection of the z-stack fluorescence images of the scaffold bearing a slope angle of (A) *ca.* 80° and (B) *ca.* 65°, respectively, after 24 h of culture. (C, D) Average fluorescence intensity distribution across the microchannel, following the path marked by the yellow dotted line in panels (A) and (B), with a sample size of 8 (N = 8). In (A) and (B), the cell nucleus was stained blue with DAPI, and the F-actin was stained red. Each microchannel is considered as an independent technical replicate.
